# Evidence of an Effect of Gaming Experience on Visuospatial Attention in Deaf but Not in Hearing Individuals

**DOI:** 10.3389/fpsyg.2020.534741

**Published:** 2020-10-20

**Authors:** Emil Holmer, Mary Rudner, Krister Schönström, Josefine Andin

**Affiliations:** ^1^Department of Behavioural Sciences and Learning, Linnaeus Centre Head, The Swedish Institute for Disability Research, Linköping University, Linköping, Sweden; ^2^Department of Linguistics, Stockholm University, Stockholm, Sweden

**Keywords:** deafness, sign language, visuospatial attention, executive function, gaming

## Abstract

Auditory cortex in congenitally deaf early sign language users reorganizes to support cognitive processing in the visual domain. However, evidence suggests that the potential benefits of this reorganization are largely unrealized. At the same time, there is growing evidence that experience of playing computer and console games improves visual cognition, in particular visuospatial attentional processes. In the present study, we investigated in a group of deaf early signers whether those who reported recently playing computer or console games (deaf gamers) had better visuospatial attentional control than those who reported not playing such games (deaf non-gamers), and whether any such effect was related to cognitive processing in the visual domain. Using a classic test of attentional control, the Eriksen Flanker task, we found that deaf gamers performed on a par with hearing controls, while the performance of deaf non-gamers was poorer. Among hearing controls there was no effect of gaming. This suggests that deaf gamers may have better visuospatial attentional control than deaf non-gamers, probably because they are less susceptible to parafoveal distractions. Future work should examine the robustness of this potential gaming benefit and whether it is associated with neural plasticity in early deaf signers, as well as whether gaming intervention can improve visuospatial cognition in deaf people.

## Introduction

Without technical intervention, congenitally profoundly deaf individuals have little opportunity to process sound. As a result, auditory cortex reorganizes to process other types of information, including visual cognition (Cardin et al., 2013, 2018; Ding et al., 2015; Twomey et al., 2017; Holmer et al., 2019; for reviews, see Alencar et al., 2019; Cardin et al., 2020), possibly offering deaf individuals the potential to outperform their hearing peers in this domain (Cardin et al., 2018). However, deaf children sometimes have difficulty achieving expected performance in academic skills, such as reading and math (Qi and Mitchell, 2012), and may not realize their potential as adults (Rudner et al., 2016). Performance on some visuospatial tasks, in particular those tapping into visuospatial perception and attentional processes, have been shown to be altered in deaf individuals (for reviews, see Bavelier et al., 2006; Rudner et al., 2009). In hearing individuals, visuospatial perception and attention have been reported to shift as a function of gaming experience (for recent meta-analyses, see Wang et al., 2016; Bediou et al., 2018; also see, Kristjánsson, 2013; Powers et al., 2013, for critical reviews). One study has also reported improved inhibition control in deaf individuals after playing a first-person shooter game one hour per day for 16 weeks (Nagendra et al., 2017). The aim of the present, cross-sectional, study was to investigate the combined effect of deafness and naturally occurring gaming experience on visuospatial attention.

Changes driven by congenitally deafness seem to be limited specifically to attentionally demanding aspects of visuospatial processing (Bavelier et al., 2006). Visual processing is supported by dorsal and visual neural streams. The dorsal visual stream supports processing of “where” a stimulus is and how it moves while the ventral stream supports identification of “what” the stimulus is. Both “what” and “where” processing becomes attentionally demanding in the presence of task-irrelevant information. Armstrong et al. (2002) reported evidence of an influence of deafness on the function of the dorsal visual stream. Effects of deafness are manifested in altered processing of motion in the visual periphery (Bavelier et al., 2001; Armstrong et al., 2002; Bosworth and Dobkins, 2002; Fine et al., 2005) as well as some aspects of peripheral attention in deaf individuals (Bavelier et al., 2001; Proksch and Bavelier, 2002; Colmenero et al., 2004; Dye et al., 2007; Hauser et al., 2007). In addition, detection of changes outside foveal vision seems to be faster in deaf than in hearing individuals (Loke and Song, 1991; Chen et al., 2006; Dye et al., 2009), suggesting that stimuli outside the fovea are more likely to challenge attentional control in deaf populations, at least when those stimuli are of relevance to solving the task (Bavelier et al., 2006; Belanger and Rayner, 2015). For the ventral stream, Armstrong et al. (2002) showed no effect of deafness, whereas others showed altered effects of deafness in both ventral and dorsal streams (Weisberg et al., 2012; Samar and Berger, 2017). Because the dorsal stream is susceptible to effects of deafness, with increased attentional resources used for processing of stimuli in the periphery, deaf individuals might perform worse than hearing individuals on visual tasks where stimuli outside the fovea need to be suppressed.

Working memory, the active storage of representations for ongoing processing, and attentional control, the selection of stimulus to focus on in processing, limits performance on cognitive tasks (Oberauer, 2019). For the processing of stimulus-rich displays and subsequentially presented stimuli, working memory is recruited and demands on attentional control are high. Although verbal working memory is similar for deaf and hearing individuals (Boutla et al., 2004; Andin et al., 2013), deaf individuals have better visuospatial working memory than hearing peers when assessed on a dynamic sequence tapping task, such as the Corsi Block-Tapping Test (Wilson et al., 1997; Geraci et al., 2008; Lauro et al., 2014). Similar results have been shown with a card-pair matching task (Rudner et al., 2016). This behavioral advantage may well reflect enhanced dorsal stream processing. On a static visual working memory task, however, the performance of deaf individuals has been reported to be worse than for hearing individuals (Lauro et al., 2014). It is likely that this reflects compromised ventral stream processing (cf. Samar and Berger, 2017).

In the Flanker task (Eriksen and Eriksen, 1974), the participant needs to suppress static distractors presented outside the fovea while making a decision on a target stimulus presented in the center of the visual field. Thus, it is a task requiring visuospatial attentional control for selective monitoring of what is visually present (Dye et al., 2007; Unsworth et al., 2015). This means that the Flanker task probably taps both dorsal and ventral visual stream functions and this notion is supported by empirical data (Lange-Malecki and Treue, 2012; Perry and Fallah, 2014; McDermott et al., 2017). A slowing of performance on the task is typically observed as the incongruence between response selection for a target stimulus and flanking distractors increases (Eriksen and Eriksen, 1974; Rueda et al., 2004; Sladen et al., 2005; Dye et al., 2007), indicating a conflict in determining what the target is. The standard task typically has two response keys, corresponding to two different targets, and incongruence is achieved by presenting flanking stimuli that correspond to the non-target response key. In other trials, flanking stimuli are congruent with the target stimulus, which leads to faster responses. The difference in response times between incongruent and congruent trials is an indicator of visuospatial attentional control (Rueda et al., 2004), and with an increase in attentional allocation to stimuli outside the fovea in deaf individuals (Bavelier et al., 2006) as well as altered ventral stream processing (Weisberg et al., 2012; Samar and Berger, 2017), an incongruence effect is likely to be stronger for deaf compared to hearing individuals. Thus, despite superior performance on some tasks related to the dorsal stream, deaf individuals are more distracted by flanking stimuli in a Flanker task than hearing participants (Dye et al., 2007; Dye and Hauser, 2014), irrespective of sign language skill (Proksch and Bavelier, 2002; Dye et al., 2007; also see Bosworth and Dobkins, 2002; Dye et al., 2009). This does, however, align with the notion of changed ventral stream processing in deaf compared to hearing individuals shown in some studies, since the Flanker task poses a challenge in maintaining control of *what* (i.e., ventral) is presented on the screen, rather than *where* (i.e., dorsal) stimuli are located.

Visuospatial attentional control is a domain that has been reported to be improved by gaming experience (Wang et al., 2016; Bediou et al., 2018). In fact, a recent meta-analysis (Bediou et al., 2018), indicated robust effects of gaming experience on top-down attentional control tasks, including Flanker tasks. Greenwood and Parasuraman (2016) argue that in the initial stages of cognitive training the dorsal stream is recruited through a bottom-up process of distraction suppression, but as the need for distraction suppression is reduced with increasing skill, functional disconnection of the dorsal stream occurs. Thus, reduced load on dorsal stream function as a result of cognitive training may make attentional resources available for transfer to other tasks. Nagendra et al. (2017) reported improved performance of deaf individuals on a Stroop color-word task, as indexed by shorter response latency, after a video gaming intervention. In a Stroop color-word task, participants have to shield themselves from interference effects when the color and the word do not match (Scarpina and Tagini, 2017), in a manner analogous to the Flanker task. However, although the Stroop task is visual, interference effects are semantico-lexical rather than visuospatial.

Previous studies on hearing populations suggest that effects of gaming experience on visuospatial attention might be restricted to specific type of games. In particular, action video games (AVGs) have been suggested to be faciliative (Wang et al., 2016; Bediou et al., 2018). AVGs are described as fast paced, to rely on flexible use of visuospatial attention, and involve dealing with a multitude of objects on screen simultaneously. However, different criteria for labeling games are used in the literature, and what qualifies as an AVG and what does not, is not easily determined (see Bediou et al., 2018). Importantly, types of games other than AVGs have also been reported to improve cognition, and it has been suggested that specific changes in cognition are to be expected for specific type of games (i.e., near-transfer effects, Oei and Patterson, 2013). This notion is similar to the idea that differences in visuospatial attention between deaf and hearing individuals are specific and experience-based (Bavelier et al., 2006; Samar and Berger, 2017). Here, we wanted to investigate this association by comparing performance on a Flanker task of deaf individuals who report they play video or computer games, to those who report that they do not play such games.

In the present study, we predict the negative effect on response times of distracting stimuli in a Flanker task to be greater for deaf than hearing individuals (see e.g., Dye et al., 2007). However, as gaming experience has been shown to improve visuospatial attentional control (Bediou et al., 2018), and gamers are expected to show less interference from incongruent flankers than non-gamers, we predict that gamers will outperform non-gamers on the Flanker task.

## Materials and Methods

### Participants

We included 16 early deaf (9 female) and 24 hearing (12 female) participants. All had normal or corrected-to-normal visual acuity and normal contrast sensitivity, as measured by Snellen chart (McGraw et al., 1995) and Pelli-Robson contrast sensitivity chart (Pelli and Robson, 1988), respectively. Due to recruitment constraints, deaf participants (*M* = 35.1, *SD* = 7.6, range 22–48) were on average almost 9 years older than the hearing participants (*M* = 26.5, *SD* = 7.5, range 19–40) and this difference was statistically significant, *t*(22.2) = 3.44, *p* = 0.002, ε = 0.64. However, there was no statistically significant difference between groups in non-verbal cognitive ability, *t*(12.3) = 0.91, *p* = 0.38, ε = 0.25, as measured on the Visual puzzles subset from WAIS-IV (Wechsler, 2008). All participants had completed at least high school (minimum of 12 years); six deaf and seven hearing participants had a university degree.

Deaf participants used Swedish Sign Language (Svenskt teckenspråk; STS) as their primary language. Nine were deaf from birth and the remaining seven were between 6 months and 3 years old when their deafness was confirmed. Five had deaf parents who signed with them from birth, and the rest started to learn sign language as soon as their deafness was discovered, and their parents started to use STS. For nine participants this was before the age of 3, and for one participant, this was in pre-school years. One participant did not specify when they started using sign language.

### Gaming Experience

To classify participants as a gamer or a non-gamer, participants answered a questionnaire (see Supplementary Appendix A; for similar procedures, see e.g. Rudner et al., 2015; Unsworth et al., 2015) on their gaming habits. Since the literature on gaming effects on visuospatial attention is limited to hearing populations, and we know little of whether reported effects generalize to deaf populations, assignment by self-report was applied instead of more extensive, and costly, longitudinal designs. Participants were asked how often (0 = Not at all, 1 = Less than once per week, 2 = One to three days per week, 3 = Four to six days per week, 4 = Every day, or 5 = Several times, every day) they had been playing computer and/or console games (including games on handheld consoles) during the last 6 months. We did not assess whether gaming intensity varied during this period, or if this period was a representative example of the individual’s general gaming pattern. Based on self-reported gaming experience, participants were then categorized as a gamer or a non-gamer. All participants who reported having played any type of game on a computer or console or both during the last six months were defined as gamers (i.e., response categories 1–5). All participants who reported not playing computer or console games at all during the last 6 months were defined as non-gamers (i.e., response category 0). Among hearing participants, 12 (2 female) were categorized as gamers and 10 (8 female) as a non-gamers (two female participants did not report gaming experience), and among deaf participants, there were 8 gamers (3 female) and 8 non-gamers (6 female). Of the deaf gamers, 4 reported playing only console games and 1 played only computer games, the rest played both, and of the hearing gamers, 5 played console games only, 3 only computer games, and the rest played both computer and console games. We did not make sub-groups based on the type of games participants played (see Supplementary Appendix B for a list of the games participants reported playing). This was partly due to the small sample size, but also because the previous literature on gaming effects almost exclusively include hearing populations.

### The Flanker Task

**FIGURE 1:**
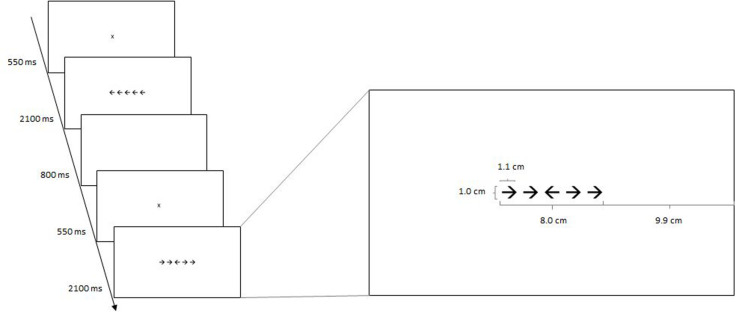
On the left hand side, an overview of the structure of the Flanker task, with examples of one congruent trial (five arrows pointing in the same direction) and one incongruent trial (four flanking arrows pointing to the right, and the middle arrow pointing to the left). To the right, a depiction of how the arrows were displayed on the screen.

In the Flanker task (Eriksen and Eriksen, 1974), participants had to decide whether a target stimulus, which was an arrow presented at the center of a computer screen (e.g., Dye et al., 2007; Unsworth et al., 2015), pointed left or right, and respond by pressing the corresponding button on the keyboard. Specifically, if the target stimulus was an arrow pointing left, the participant was instructed to press the left Shift key (marked with an arrow pointing to the left drawn on a piece of self-adhesive paper) and if the target stimulus was an arrow pointing right, the participant was instructed to press the right Shift key (marked with an arrow pointing to the right drawn on a piece of self-adhesive paper). In each trial, the target stimulus was flanked by two arrows on each side. Congruent trials had flankers pointing in the same direction as the target (e.g.,←←←←←) and incongruent trials, in the opposite direction (e.g.,←←→←←). The participant was instructed to ignore the flanker arrows and respond to the direction of the target arrow. A trial began with a fixation point presented in the middle of the screen for 550 ms, which was immediately followed by a horizontal array, 8 cm wide, of five equally sized and equally spaced black arrows. The array remained on the screen for 2100 ms, after which the screen went blank for 800 ms before the start of the next trial. For an overview of the structure of the task, see Figure 1. The task was administered on a 12″ laptop computer using presentation software DMDX version 5.1.4.2 (Forster and Forster, 2003) and the distance between the participant’s face and the screen was approximately 60 cm. Participants responded to 48 trials in total, with an equal number of congruent and incongruent trials. In half of the trials within each condition, the target pointed to the left, and in the other half to the right. The order of presentation was randomized for each participant. The dependent variable was average response time in ms on trials to which a correct response was given (both for congruent and incongruent trials).

### Swedish Sign Language Sentence Repetition Test

To rule out inadequate sign language skills as an explanation for the results in the present study, deaf participants’ STS skill was assessed on the Swedish Sign Language Sentence Repetition Test (STS-SRT, Schönström, 2014a,b). The STS-SRT is an adaptation of an American Sign Language sentence repetition test (ASL-SRT, Hauser et al., 2008) used to measure global sign language fluency of deaf adults. The STS-SRT is a reliable and valid test of STS skills in adults who have used STS since childhood (Schönström, 2014b). The test consisted of 31 trials with filmed STS sentences produced by a deaf native signing man. The sentences varied in length and in difficulty. The participant was instructed to watch the sentences and to reproduce them exactly as signed in the video clips, including the vocabulary and grammatical markers used. Before testing started, participants practiced on three sentences to make sure that they had understood the procedure. On each trial in the actual test, the participants saw a video clip presented on a laptop (12′′ screen), and were given approximately 8 seconds to repeat the sentence before the next trial started. The front camera on the laptop was used to film responses. Responses were scored based on a guideline with instructions for each trial on a later occasion (Schönström, 2014b). For a response to be scored as correct, the participants had to reproduce the sentence exactly as it was performed. The dependent variable was number of correctly reproduced sentences (maximum = 31). Testing time was approximately 10 minutes.

### Procedure

Participants were tested individually in a quiet room. Participants provided written informed consent before behavioral testing commenced. This study is part of a larger project and testing started with screening of visual acuity and visual contrast, before a cognitive test battery, including tests of episodic long-term memory, lip-reading ability, and phonological skill, in addition to the test of non-verbal cognitive ability (Visual puzzles, Wechsler, 2008), STS skill (STS-SRT, Schönström, 2014a) and the Flanker task reported here, was administered. Before the test battery was administered, participants performed one motor speed task and a physical matching task (Holmer et al., 2016) to become familiar with the set-up of the computerized testing. Testing took approximately 60 minutes in total. For deaf participants, an accredited STS interpreter was present during testing and provided verbatim translation of instructions. In a second part of the larger project, participants performed an fMRI experiment not reported here.

### Statistical Analysis

First, descriptive statistics and frequencies for control and background variables were calculated, and the distribution of response times from the Flanker task were visually inspected. Due to the small sample size with associated potential threats of non-normality and low power, robust statistical methods were applied (Erceg-Hurn and Mirosevich, 2008; Wilcox, 2017). Statistical analysis was performed in RStudio version 1.2.5042 (RStudio Team, 2020), running R version 4.0.0 (R Core Team, 2020). Group comparisons on control and background variables: age, non-verbal cognitive ability, STS skill (only deaf participants), and gaming habits for gamers, were performed using yuen *t*-tests with the *yuen* function from package WRS2 (Mair and Wilcox, 2020). As an estimate of effect size the explanatory measure of effect size ε is reported, with values of 0.1, 0.3, and 0.5 corresponding to small, medium, and large effects (Mair and Wilcox, 2020). After that, Wilcox (2017) *bbwtrim* function was used to perform a robust mixed ANOVA with one within-group factor: Congruency (congruent, incongruent), and two between-group factors: Group (deaf, hearing) and Gaming (gamer, non-gamer), on response time (in ms) from the Flanker task. Effect size estimates ε for main effects of the ANOVA were calculated with the *yuen* function for between group effects and the *yuend* function for the within group effect, both from package WRS2 (Mair and Wilcox, 2020). Main effects were followed up by comparing means between levels of the factor, and simple main effects were followed up by comparing percentile bootstrapped confidence intervals, estimated using the *onesampb* function from WRS2 (Mair and Wilcox, 2020). To investigate associations between age and non-verbal cognitive ability and performance on the Flanker task, robust correlations were calculated with the *pbcor* function from WRS2 (Mair and Wilcox, 2020). The default value of a trim proportion of 0.2 was applied in all robust analyses. Due to a technical issue, the result was missing for one deaf non-gamer on the Flanker task. One hearing gamer and one hearing non-gamer performed on chance level on the Flanker task, indicating that they did not follow instructions. The mean performance of the sub-group that the participant belonged to was used for these three participants in analyses to maximize statistical power.

## Results

### Characteristics of Deaf and Hearing Gamers and Non-gamers

Descriptive statistics on background variables for deaf and hearing gamers and non-gamers are reported in Table 1. Deaf participants demonstrated proficiency in STS skills, as assessed on the STS-SRT (mean performance was on par with mean performance from a previously tested group, *M* = 17.7 och SD = 4.9, Schönström, 2014a,b). No statistically significant differences on any background variables were seen between deaf gamers and non-gamers: age, *t*(6.6) = 0.00, *p* = 1.00, ε = 0.00, non-verbal cognitive ability, *t*(8.7) = 2.08, *p* = 0.07, ε = 0.61, and STS skill, *t*(10) = 0.67, *p* = 0.52, ε = 0.31. Similarly, hearing gamers and non-gamers did not differ on background variables: age, *t*(8.7) = 0.00, *p* = 1.00, ε = 0.07, and visual puzzles, *t*(11.6) = 0.26, *p* = 0.80, ε = 0.13. Thus, there were no underlying differences on background variables between gamers and non-gamers in either of the two groups.

**TABLE 1:** Descriptive statistics on background variables for deaf and hearing gamers and non-gamers.

Variable	Deaf				Hearing			
		
	Gamer (*n* = 8)		Non-gamer (*n* = 8)		Gamer (*n* = 12)		Non-gamer (*n* = 10)	
				
	*M*	*SD*	*M*	*SD*	*M*	*SD*	*M*	*SD*
Age	34.9	4.79	35.4	10.0	25.9	7.04	26.3	7.70
VP	12.8	4.06	9.63	2.39	12.5	2.88	12.5	1.96
STS-SRT	16.8	3.96	18.6	4.21				

*VP = Visual puzzles, standardized score; STS-SRT = Swedish Sign Language-Sentence Reception Test, raw score.*

To compare gaming habits of deaf and hearing gamers, ratings on how often they played computer respectively console games were compared. Groups reported similar gaming habits; for computer games, deaf gamers (*M* = 0.63, *SD* = 0.74) compared to hearing gamers (*M* = 1.08, *SD* = 1.24), *t*(11.3) = 0.79, *p* = 0.45, ε = 0.27, and for console games, deaf gamers (*M* = 1.50, *SD* = 1.00) compared to hearing gamers (*M* = 0.92, *SD* = 0.67), *t*(10.4) = 1.43, *p* = 0.18, ε = 0.13.

### Flanker Task

**FIGURE 2:**
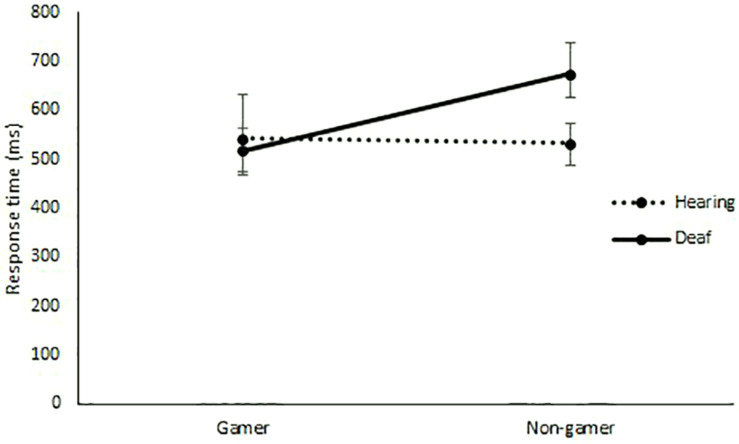
Response time (in ms, *y*-axis) for gamers and non-gamers (*x*-axis) for deaf and hearing participants. Error bars represents 95% confidence intervals.

As expected, deaf gamers (*M* = 98%, *SD* = 5.8) and non-gamers (*M* = 98%, *SD* = 4.2), as well as hearing gamers (*M* = 99%, *SD* = 1.4, after exclusion of the participant who performed at chance level) and non-gamers (*M* = 99%, *SD* = 2.3, after exclusion of the participant who performed at chance level) performed close to ceiling on accuracy on the Flanker task. Thus, response times for almost all trials were included in the analysis (see Table 2 for descriptive statistics). The mixed robust ANOVA for response times in Flanker showed a main effect of congruency, *Q* = 74.1, *p* < 0.001, ε = 0.32, gaming, *Q* = 5.40, *p* = 0.02, ε = 0.41, and of Group, *Q* = 5.09, *p* = 0.02, ε = 0.41. Response time was faster for congruent (*M* = 539 ms, *SD* = 110) than incongruent (*M* = 597, *SD* = 114) trials, and gamers (*M* = 541 ms, *SD* = 112) responded faster than non-gamers (*M* = 598 ms, *SD* = 102), and hearing (*M* = 557 ms, *SD* = 108) responded faster than deaf (*M* = 594 ms, *SD* = 108). There was a statistically significant interaction between group and gaming, *Q* = 8.89, *p* = 0.003 (see Figure 2). Investigation of the confidence intervals for the group by gamer interaction, indicated that deaf gamers, 95% CI [475 ms, 562 ms], responded faster than deaf non-gamers, 95% CI [626 ms, 739 ms], and on par with hearing gamers, 95% CI [468 ms, 631 ms]. Hearing non-gamers, [486 ms, 573 ms], responded faster than deaf non-gamers, but no difference was observed in comparison to hearing gamers. Thus, the main effect of gaming experience was explained by a group-specific effect for deaf participants that eliminated any difference in processing efficiency across groups.

**TABLE 2:** Response times (mean, median, and standard deviation) for deaf and hearing gamers and non-gamers on congruent and incongruent trials in the Flanker task.

Trial type	Deaf						Hearing					
		
	Gamer (*n* = 8)			Non-gamer (*n* = 8)			Gamer (*n* = 12)			Non-gamer (*n* = 10)		
				
	*M*	*Mdn*	*SD*	*M*	*Mdn*	*SD*	*M*	*Mdn*	*SD*	*M*	*Mdn*	*SD*
Congruent	485	496	67	644	633	33	528	496	144	511	498	64
Incongruent	553	536	61	720	711	46	583	554	132	551	538	61

Besides the interaction between Group and Gaming, interactions were not statistically significant (all *p*s > 0.05). Thus, our predictions that deaf individuals are more distracted and that gamers are less distracted by incongruent flanking stimuli were not supported. Non-verbal cognitive ability, *r*_*pb*_ = −0.21, *p* = 0.19, and age, *r*_*pb*_ = 0.23, *p* = 0.16, were not associated with response time on the Flanker task, and it is thus unlikely that these variables strongly influenced the pattern of results.

## Discussion

In the present study, we investigated the effect of naturally occurring gaming experience on visuospatial attentional control in early deaf signers. We predicted longer response times on the Flanker task for deaf compared to hearing participants and that this difference would be most apparent for incongruent trials. We also predicted that gamers would show less interference from flankers than non-gamers and outperform them on the Flanker task, especially for incongruent trials.

Our predictions were partially supported by the results. While both deaf and hearing groups had longer response times on incongruent than congruent trials, the deaf group did not show longer response times than the hearing group specifically on incongruent trials. Instead, the deaf group responded slower on both congruent and incongruent trials. Across groups, deaf non-gamers responded slower than hearing non-gamers, while there was no significant difference in performance between deaf gamers and hearing participants. Further, an effect of gaming was only observed in the deaf group, and we did not find evidence of a specific effect of gaming on incongruent trials.

Although there was a statistically significant main effect of group on performance on the Flanker task, this effect was explained by longer latencies for deaf non-gamers compared to the other participants. Deaf gamers performed similar to hearing participants. With enhanced visuospatial perception in deaf compared to hearing participants under some circumstances (Loke and Song, 1991; Chen et al., 2006; Dye et al., 2009), worse performance on tasks demanding control of visuospatial attention might seem contradictory. However, these seemingly contradictory findings might be explained by differences in ventral versus dorsal stream processing, and their relative contribution to the behavioral task (Samar and Berger, 2017). Proksch and Bavelier (2002) proposed that congenital deafness alters visuospatial attention in such a way that more attentional resources are used for processing stimuli outside central vision (also, see Bavelier et al., 2006). In a visuospatial perception task designed to invoke dorsal stream functions, this will lead to better ability to, e.g., detect stimuli in the periphery (e.g., Dye et al., 2009), but in a task that relies more on ventral stream processing, and suppression of dorsal stream elements, performance might be impaired (e.g., Dye et al., 2007). Like Lauro et al. (2014), here we used a static task that could be argued to rely on ventral stream processing, and in line with what Lauro et al. (2014) reported, we saw worse performance in deaf compared to hearing individuals. Thus, our results lend further behavioral support to the notion of potentially altered ventral stream processing in deaf populations (Weisberg et al., 2012; Samar and Berger, 2017). On the other hand, we did not find evidence that deaf participants are more distracted by incongruent flanking stimuli than hearing participants. In line with previous data (e.g., Dye et al., 2007), we reasoned that the effect of incongruency would become stronger as a consequence of the redistribution of visuospatial attention. It is likely that the small sample in combination with the complexity of the design might have been at play here. To maximize power and minimize bias due to potential non-normality in the data, robust methods were used in analysis. Although this was likely to be the best analytic approach for the purposes of the present study, the results are still constrained by the available data. In addition to the limited amount of individuals, the Flanker task only included 24 congruent and 24 incongruent trials. This number of trials is similar to what others have used (i.e., 30 for each type in Unsworth et al., 2015), but more trials are likely to produce more stable estimates when averaging within individual, with reduced noise in the analysis as a result (Brysbaert, 2019). These factors: small sample, complex design, and small number of trials, are likely to have reduced the probability of detecting a group by congruency interaction. Thus, we cannot rule out the possibility that deaf individuals are more distracted by incongruent flanking stimuli in a Flanker task than hearing individuals.

Based on the present study we suggest that deaf individuals with recent gaming experience reveal a level of visuospatial attentional control similar to that revealed by hearing individuals in a task that presumably draws upon ventral stream processing. To our knowledge, only one previous study has investigated effects of gaming on cognition in a deaf population (on a Stroop color-word task, Nagendra et al., 2017), and that study also reported a positive effect. Our findings extend the results of Nagendra et al.’s (2017) study, by showing an effect of gaming in another executive domain. Importantly, the effect of gaming in the present study was not simply driven by sign language proficiency, since sign language skills did not differ between deaf gamers and non-gamers. Greenwood and Parasuraman (2016) argue that cognitive training leads to functional disconnection of the dorsal stream, releasing attentional resources for transfer to other tasks. Because a specific effect of gaming is found only for deaf individuals with potentially enhanced dorsal stream skills, one interpretation is that this group has more resources to transfer as a result of the cognitive training inherent in gaming. A potential group-specific effect of gaming experience in deaf individuals needs to be followed up in future work. In particular, combining behavioral and brain imaging measures will help us illuminate potential alterations in dorsal and/or ventral stream processing. Related to this, an effect should also be compared between congenitally deaf individuals and individuals with acquired deafness.

Previous studies in hearing individuals have reported effects of gaming on the kind of attentional control demanded by a Flanker task (Bediou et al., 2018). However, here we did not see any effect of gaming in the hearing group, and there was no significant interaction between gaming and congruency. Although it might be the case, as some argue, that gaming experience does not lead to any meaningful effects on cognitive functions in hearing individuals (Kristjánsson, 2013; Powers et al., 2013), the present study had some limitations that might explain why our results were not in line with our prediction. As already mentioned, statistical power was restricted due to the small sample size, another issue might be that our definition of a gamer was not as strict as definitions applied in previous studies in the literature (e.g., Bediou et al., 2018). Further, self-reported gaming habits during the last six months determined group assignment. In hearing individuals, there is evidence to suggest that gaming effects vary as a function of gaming genre (however, see a discussion on issues in defining genres in Bediou et al., 2018). In particular, action video games (AVGs) seem to have the most robust effects (Wang et al., 2016; Bediou et al., 2018). Gamers in the present study played a wide variety of games (see Supplementary Appendix B), ranging from simple puzzle games (not typically categorized as AVGs, e.g., Tetris) to first-person shooters (commonly categorized as AVGs, e.g., Counter-strike), and there was also variability in what type of platform they preferred for playing games (i.e., some played games on stationary consoles, others on a computer, and yet others on both these types of platforms). Self-report measures are convenient, but they do not always reflect actual behavior, and this is true also in the case of gaming experience (Kahn et al., 2014). Besides the potentially low correspondence to actual behavior, the temporal resolution of the self-report measure included here was coarse. It is possible that effects of video games on visuospatial attention are transient (similar to effects of gaming on attitudes, e.g., Sestir and Barthalow, 2010), which might have then influenced our results. As two examples, we do not know whether participants in one group had more recent gaming experience than the participants in the other group, or if participants had played for only a limited period during the time for which they reported their habits. Our approach was, however, intentional and motivated by a number of factors. Most importantly, we did not find any previous study on the effect of gaming experience on visuospatial attention in deaf individuals, but plenty of evidence to suggest that visuospatial processing differs between deaf and hearing individuals (Bavelier et al., 2006). Thus, we had little reason to assume that findings from hearing populations would be exactly the same for deaf individuals. However, since we did find an effect in deaf individuals, and saw that groups reported similar gaming habits, this could mean that effects of gaming experience on visuospatial attentional control are observed with a lower dose of exposure in this population. One explanation for this could be that the mechanisms are somewhat different across groups, and more malleable to visuospatial experience for deaf individuals. It is reasonable to assume that effects arising from gaming experience are constrained by baseline levels across tasks, and with different baselines in visuospatial attention across deaf and hearing populations, the pattern across groups is influenced by task selection. Oei and Patterson (2013) suggest that game characteristics constrain transfer, and here we propose that the characteristics of the gamer will produce similar constraints. It is thus important to further investigate the role of different types of gaming experiences in visuospatial perception, and visuospatial attention in particular, in deaf individuals. Experimental designs are a way forward, with active manipulation of gaming experience, although that might become more and more challenging with gaming turning into a mainstream leisure activity in society. As an alternative, using fine-grained correlational approach, for example, by following participants over a longer period of time and using active measures of gaming experience, such as ecological momentary assessment (Kirchner and Shiffman, 2008), might be useful in future studies. Also, the longevity of gaming effects on cognition is something that needs to be addressed in such work.

## Conclusion

Visuospatial attention is altered by early deafness. The results of the present study show better visuospatial attentional control in deaf signers who play video games than those who do not. Gaming experience may help harness the changes in visuospatial attention displayed by deaf individuals for better attentional control. Thus, gaming might be a useful intervention for shielding deaf children from potential visuospatial distractions.

## Data Availability Statement

All datasets generated for this study are included in the article/Supplementary Material.

## Ethics Statement

The studies involving human participants were reviewed and approved by the Regional Ethical Review Board, Linköping, Sweden (dnr 2016/344-31). The participants provided their written informed consent to participate in this study.

## Author Contributions

EH, MR, and JA designed the study. JA collected data. KS scored performance on the STS-SRT. EH performed the data analysis. All authors were involved in the interpretation of results as well as preparing and finalizing the manuscript, after a draft version was prepared by EH.

## Conflict of Interest

The authors declare that the research was conducted in the absence of any commercial or financial relationships that could be construed as a potential conflict of interest.

## References

[B1] Alencar C. D. C. Butler B. E. Lomber S. G. (2019). What and how the deaf brain sees. *J. Cogn. Neurosc.* 31 1091–1109. 10.1162/jocn 31112472

[B2] Andin J. Orfanidou E. Cardin V. Holmer E. Capek C. M. Woll B. (2013). Similar digit-based working memory in deaf signers and hearing non-signers despite digit span differences. *Front. Psychol.* 4:942. 10.3389/fpsyg.2013.00942 24379797 PMC3863759

[B3] Armstrong B. A. Neville H. J. Hillyard S. A. Mitchell T. V. (2002). Auditory deprivation affects processing of motion, but not color. *Cogni. Brain Res.* 14 422–434. 10.1016/s0926-6410(02)00211-2 12421665

[B4] Bavelier D. Brozinsky C. Tomann A. Mitchell T. Neville H. Liu G. (2001). Impact of early deafness and early language exposure to sign language on the cerebral organization for motion processing. *J. Neurosci.* 21 8931–8942. 10.1523/JNEUROSCI.21-22-08931.2001 11698604 PMC6762265

[B5] Bavelier D. Dye M. W. G. Hauser P. C. (2006). Do deaf individuals see better? *Trends Cogn. Sci.* 10 512–518. 10.1016/j.tics.2006.09.006 17015029 PMC2885708

[B6] Bediou B. Mayer R. Barbara S. Tipton E. Bavelier D. (2018). Meta-analysis of action video game impact on perceptual, attentional, and cognitive skills. *Psychol. Bull.* 144 77–110. 10.1037/bul0000130 29172564

[B7] Belanger N. N. Rayner K. (2015). What eye movements reveal about deaf readers. *Curr. Dir. Psychol. Sci.* 24 220–226. 10.1177/0963721414567527 26594098 PMC4651440

[B8] Bosworth R. G. Dobkins K. R. (2002). The effects of spatial attention on motion processing in deaf signers, hearing signers, and hearing nonsigners. *Brain Cogn.* 49 152–169. 10.1006/brcg.2001.1497 12027400

[B9] Boutla M. Supalla T. Newport E. L. Bavelier D. (2004). Short-term memory span: insights from sign language. *Nat. Neurosci.* 7 997–1002. 10.1038/nn1298 15311279 PMC2945821

[B10] Brysbaert M. (2019). How many participants do we have to include in properly powered experiments? A tutorial of power analysis with reference tables. *J. Cogn.* 2:16. 10.5334/joc.72 31517234 PMC6640316

[B11] Cardin V. Grin K. Vinogradova V. Manini B. (2020). Crossmodal reorganisation in deafness: mechanisms for functional preservation and functional change. *Neurosci. Biobehav. Rev*. 113, 227–237. 10.1016/j.neubiorev.2020.03.019 32199886

[B12] Cardin V. Orfanidou E. Rönnberg J. Capek C. M. Rudner M. Woll B. (2013). Dissociating cognitive and sensory neural plasticity in human superior temporal cortex. *Nat. Commun.* 4:1473. 10.1038/ncomms2463 23403574

[B13] Cardin V. Rudner M. De Oliveira R. F. Andin J. Su M. T. Beese L. (2018). The organization of working memory networks is shaped by early sensory experience. *Cereb. Cortex* 28 3540–3554. 10.1093/cercor/bhx222 28968707

[B14] Chen Q. Zhang M. Zhou X. (2006). Effects of spatial distribution of attention during inhibition of return (IOR) on flanker interference in hearing and congenitally deaf people. *Brain Res.* 1109 117–127. 10.1016/j.brainres.2006.06.043 16859649

[B15] Colmenero J. M. Catena A. Fuentes L. J. Ramos M. M. (2004). Mechanisms of visuospatial orienting in deafness. *Eur. J. Cogn. Psychol.* 16 791–805. 10.1080/09541440340000312

[B16] Ding H. Qin W. Liang M. Ming D. Wan B. Li Q. (2015). Cross-modal activation of auditory regions during visuo-spatial working memory in early deafness. *Brain* 138 2750–2765. 10.1093/brain/awv165 26070981

[B17] Dye M. W. G. Baril D. E. Bavelier D. (2007). Which aspects of visual attention are changed by deafness? The case of the attentional network test. *Neuropsychologia* 45 1801–1811. 10.1038/jid.2014.371 17291549 PMC2885017

[B18] Dye M. W. G. Hauser P. C. (2014). Sustained attention, selective attention and cognitive control in deaf and hearing children. *Hearing Res.* 309 94–102. 10.1016/j.heares.2013.12.001 24355653 PMC3928356

[B19] Dye M. W. G. Hauser P. C. Bavelier D. (2009). Is visual selective attention in deaf individuals enhanced or deficient? The case of the useful field of view. *PLoS One* 4:5640. 10.1371/journal.pone.0005640 19462009 PMC2680667

[B20] Erceg-Hurn D. M. Mirosevich V. M. (2008). Modern robust statistical methods: an easy way to maximize the accuracy and power of your research. *Am. Psychol.* 63 591–601. 10.1037/0003-066X.63.7.591 18855490

[B21] Eriksen B. A. Eriksen C. W. (1974). Effects of noise letters upon the identification of a target letter in a nonsearch task. *Percept. Psychophys.* 16 143–149. 10.3989/arbor.2000.i650.965

[B22] Fine I. Finney E. M. Boynton G. M. Dobkins K. R. (2005). Comparing the effects of auditory deprivation and sign language within the auditory and visual cortex. *J. Cogn. Neurosci.* 17 1621–1637. 10.1162/089892905774597173 16269101

[B23] Forster K. I. Forster J. C. (2003). DMDX: a windows display program with millisecond accuracy. *Behav. Res. Methods Instrum. Comput.* 35 116–124. 10.3758/BF03195503 12723786

[B24] Geraci C. Gozzi M. Papagno C. Cecchetto C. (2008). How grammar can cope with limited short-term memory: simultaneity and seriality in sign languages. *Cognition* 106 780–804. 10.1016/j.cognition.2007.04.014 17537417

[B25] Greenwood P. M. Parasuraman R. (2016). The mechanisms of far transfer from cognitive training: review and hypothesis. *Neuropsychology* 30 742–755. 10.1037/neu0000235 26569030

[B26] Hauser P. C. Dye M. W. G. Boutla M. Green C. S. Bavelier D. (2007). Deafness and visual enumeration: not all aspects of attention are modified by deafness. *Brain Res.* 1153 178–187. 10.1016/j.brainres.2007.03.065 17467671 PMC1934506

[B27] Hauser P. C. Paludnevièiene R. Supalla T. Bavelier D. (2008). “American sign language-sentence reproduction test: development and implications,” in *Sign Language: Spinning and Unraveling the Past, Present and Future*, ed. de Quadros R. M. (Petropolis: Editora Arara Azul), 160–172.

[B28] Holmer E. Andin J. Rudner M. (2019). “Cross-modal plasticity in secondary auditory cortex,” in *11th Annual Meeting of the Society for the Neurobiology of Language*, Helsinki.

[B29] Holmer E. Heimann M. Rudner M. (2016). Evidence of an association between sign language phonological awareness and word reading in deaf and hard-of-hearing children. *Res. Dev. Disabil.* 48 145–159. 10.1016/j.ridd.2015.10.008 26561215

[B30] Kahn A. S. Ratan R. Williams D. (2014). Why we distort in self-report: predictors of self-report errors in video game play. *J. Comput. Med. Commun.* 19 1010–1023. 10.1111/jcc4.12056

[B31] Kirchner T. R. Shiffman S. (2008). Ecological momentary assessment. *Annu. Rev. Clin. Psychol.* 4 1–32. 10.1146/annurev.clinpsy.3.022806.091415 18509902

[B32] Kristjánsson Á (2013). The case for causal influences of action videogame play upon vision and attention. *Attent. Percept. Psychophys.* 75 667–672. 10.3758/s13414-013-0427-z 23386038

[B33] Lange-Malecki B. Treue S. (2012). A flanker effect for moving visual stimuli. *Vis. Res.* 62 134–138. 10.1016/j.visres.2012.03.016 22811985

[B34] Lauro L. J. R. Crespi M. Papagno C. Cecchetto C. (2014). Making sense of an unexpected detrimental effect of sign language use in a visual task. *J. Deaf Stud. Deaf Educ.* 19 358–365. 10.1093/deafed/enu001 24737843

[B35] Loke W. H. Song S. (1991). Central and peripheral visual processing in hearing and nonhearing individuals. *Bull. Psychon. Soc.* 29 437–440. 10.3758/BF03333964

[B36] Mair P. Wilcox R. (2020). Robust statistical methods in R using the WRS2 Package. *Behav. Res. Methods* 52 464–488. 10.3758/s13428-019-01246-w 31152384

[B37] McDermott T. J. Wiesman A. I. Proskovec A. L. Heinrichs-Graham E. Wilson T. W. (2017). Spatiotemporal oscillatory dynamics of visual selective attention during a flanker task. *NeuroImage* 156 277–285. 10.1016/j.neuroimage.2017.05.014 28501539 PMC5548621

[B38] McGraw P. Winn B. Whitaker D. (1995). Reliability of the Snellen chart. *BMJ Clin. Res.* 310 1481–1482. 10.1136/bmj.310.6993.1481 7787581 PMC2549871

[B39] Nagendra H. Kumar V. Mukherjee S. (2017). Evaluation of cognitive behavior among deaf subjects with video game as intervention. *Cogn. Syst. Res.* 42 42–57. 10.1016/j.cogsys.2016.11.007

[B40] Oberauer K. (2019). Working memory and attention – A conceptual analysis and review. *J. Cogn.* 2 1–23. 10.5334/joc.58 31517246 PMC6688548

[B41] Oei A. C. Patterson M. D. (2013). Enhancing cognition with video games: a multiple game training study. *PLoS One* 8:e58546. 10.1371/journal.pone.0058546 23516504 PMC3596277

[B42] Pelli D. Robson J. (1988). *The Design of a New Letter Chart for Measuring Contrast Sensitivity.* Halifax, NS: Clinical Vision Sciences.

[B43] Perry C. J. Fallah M. (2014). Feature integration and object representations along the dorsal stream visual hierarchy. *Front. Comput. Neurosci.* 8:84. 10.3389/fncom.2014.00084 25140147 PMC4122209

[B44] Powers K. L. Brooks P. J. Aldrich N. J. Palladino M. A. Alfieri L. (2013). Effects of video-game play on information processing: a meta-analytic investigation. *Psychon. Bull. Rev.* 20 1055–1079. 10.3758/s13423-013-0418-z 23519430

[B45] Proksch J. Bavelier D. (2002). Changes in the spatial distribution of visual attention after early deafness. *J. Cogn. Neurosci.* 14 687–701. 10.1162/08989290260138591 12167254

[B46] Qi S. Mitchell R. E. (2012). Large-scale academic achievement testing of deaf and hard-of-hearing students: past, present, and future. *J. Deaf Stud. Deaf Educ.* 17 1–18. 10.1093/deafed/enr028 21712463

[B47] R Core Team (2020). *R: A Language and Environment for Statistical Computing.* Vienna: R Core Team.

[B48] RStudio Team (2020). *RStudio: Integrated Development for R.* Boston: RStudio, Inc.

[B49] Rudner M. Andin J. Rönnberg J. (2009). Working memory, deafness and sign language. *Scand. J. Psychol.* 50 495–505. 10.1111/j.1467-9450.2009.00744.x 19778397

[B50] Rudner M. Keidser G. Hygge S. Rönnberg J. (2016). Better visuospatial working memory in adults who report profound deafness compared to those with normal or poor hearing: data from the UK Biobank resource. *Ear Hear.* 37 620–622. 10.1097/AUD.0000000000000314 27232076

[B51] Rudner M. Toscano E. Holmer E. (2015). Load and distinctness interact in working memory for lexical manual gestures. *Front. Psychol.* 6:1147. 10.3389/fpsyg.2015.01147 26321979 PMC4535352

[B52] Rueda M. R. Fan J. McCandliss B. D. Halparin J. D. Gruber D. B. Lercari L. P. (2004). Development of attentional networks in childhood. *Neuropsychologia* 42 1029–1040. 10.1016/j.neuropsychologia.2003.12.012 15093142

[B53] Samar V. J. Berger L. (2017). Does a flatter general gradient of visual attention explain peripheral advantages and central deficits in deaf adults? *Front. Psychol.* 8:713. 10.3389/fpsyg.2017.00713 28559861 PMC5433326

[B54] Scarpina F. Tagini S. (2017). The stroop color and word test. *Front. Psychol.* 8:557. 10.3389/fpsyg.2017.00557 28446889 PMC5388755

[B55] Schönström K. (2014a). “Adaptation of sign language tests,” in *36th Language Testing Research Colloquium (LTRC)*, Amsterdam.

[B56] Schönström K. (2014b). *Swedish Sign Language Sentence Reproduction Test.* Stockholm: Stockholm University.

[B57] Sestir M. A. Barthalow B. D. (2010). Violent and nonviolent video games produce opposing effects on aggressive and prosocial outcomes. *J. Exp. Soc. Psychol.* 46 934–942. 10.1016/j.jesp.2010.06.005

[B58] Sladen D. P. Tharpe A. M. Ashmead D. H. Grantham D. W. Chun M. M. (2005). Visual attention in deaf and normal hearing adults: effects of stimulus compatibility. *J. Speech Lang. Hear. Res.* 48 1529–1537. 10.1044/1092-4388(2005/106) 16478388

[B59] Twomey T. Waters D. Price C. J. Evans S. Macsweeney M. (2017). How auditory experience differentially influences the function of left and right superior temporal cortices. *J. Neurosci.* 37 9564–9573. 10.1523/JNEUROSCI.0846-17.2017 28821674 PMC5618270

[B60] Unsworth N. Redick T. S. McMillan B. D. Hambrick D. Z. Kane M. J. Engle R. W. (2015). Is playing video games related to cognitive abilities? *Psychol. Sci.* 26 759–774. 10.1177/0956797615570367 25896420

[B61] Wang P. Liu H. H. Zhu X. T. Meng T. Li H. J. Zuo X. N. (2016). Action video game training for healthy adults: a meta-analytic study. *Front. Psychol.* 7:907. 10.3389/fpsyg.2016.00907 27378996 PMC4911405

[B62] Wechsler D. (2008). *Wechsler Adult Intelligence Scale*, 4th Edn San Antonio, TX: Pearson Assessment.

[B63] Weisberg J. Koo D. S. Crain K. L. Eden G. F. (2012). Cortical plasticity for visuospatial processing and object recognition in deaf and hearing signers. *NeuroImage* 60 661–672. 10.1016/j.neuroimage.2011.12.031 22210355 PMC3288167

[B64] Wilcox R. (2017). *Introduction to Robust Estimation and Hypothesis Testing*, 4th Edn Amsterdam: Academic Press.

[B65] Wilson M. Bettger J. Niculae I. Klima E. (1997). Modality of language shapes working memory: evidence from digit span and spatial span in ASL signers. *J. Deaf Stud. Deaf Educ.* 2 150–160. 10.1093/oxfordjournals.deafed.a014321 15579844

