# Supplementary material for: Evidence of an Effect of Gaming Experience on Visuospatial Attention in Deaf but Not in Hearing Individuals
Source: Front Psychol. 2020 Oct 20;11:534741. doi: 10.3389/fpsyg.2020.534741 (PMC7606995; doi:10.3389/fpsyg.2020.534741)
Supplement: Supplementary file 2 [file Data_Sheet_2.PDF]

Gaming habits, past 6 months

Research suggests that people who play video and computer games might improve their memory skills. Here, we want you to answer a few questions about your gaming habits. When responding to the questions, think about the past 6 months.

- * During the last 6 months, how often did you play games on...?
    - ...a computer
    - ...a stationary console (e.g. Xbox, Playstation, Wii U)
    - ...a handheld console (t.ex. Nintendo 3DS, PS Vita)

  Alternatives:

    - (5) Several times every day
    - (4) Every day
    - (3) 4-6 days per week
    - (2) 1-3 days per week
    - (1) Less than once per week
    - (0) Not at all

- Which games did you play on a computer during the last six months? Respond with the titles of the games. Titles are separated with comma.
- Which games did you play on a stationary console during the last six months? Respond with the titles of the games. Titles are separated with comma.
- Which games did you play on a handheld console during the last six months? Respond with the titles of the games. Titles are separated with comma.

- Thinking about a gaming session, how long does it typically last...? (one response per platform)

    - ...on a computer
    - ...on a stationary console
    - ...on a handheld console

  Alternatives:

    - (4) More than 5 hours
    - (3) 3-4 hours
    - (2) 1-2 hours
    - (1) Less than 1 hour
