# Supplementary material for: Evidence of an Effect of Gaming Experience on Visuospatial Attention in Deaf but Not in Hearing Individuals
Source: Front Psychol. 2020 Oct 20;11:534741. doi: 10.3389/fpsyg.2020.534741 (PMC7606995; doi:10.3389/fpsyg.2020.534741)
Supplement: Supplementary file 3 [file Data_Sheet_3.PDF]

```
#R code for running robust analyses on the data in Evidence of an effect of gaming experience on visuospatial attention in deaf but not in hearing individuals

#special package used in analysis
install.packages("WRS2")
library(WRS2)

set.seed(99)

#Importing data set
gaming_wide <- read.csv2("C:/ADD PATH/Gaming_deafness_visuospatial_attention_data.csv")

#run code and open rallfun-v37.txt (download from https://dornsife.usc.edu/labs/rwilcox/software/) when the dialogue box appears to load Wilcox (2017) functions (used below)
source(file.choose())

#Creating sub-Groups
gaming_deaf <- gaming_wide[which(gaming_wide$Group==1), ]
gaming_hear <- gaming_wide[which(gaming_wide$Group==2), ]
gamers <- gaming_wide[which(gaming_wide$Gaming==1), ]

#Tests on background variables
WRS2::yuen(Age ~ Group, data =gaming_wide)
WRS2::yuen(Visual_puzzles_norm ~ Group, data =gaming_wide)

WRS2::yuen(Age ~ Gaming, data = gaming_deaf)
WRS2::yuen(STS_SRT ~ Gaming, data = gaming_deaf)
WRS2::yuen(Visual_puzzles_norm ~ Gaming, data = gaming_deaf)

WRS2::yuen(Age ~ Gaming, data = gaming_hear)
WRS2::yuen(Visual_puzzles_norm ~ Gaming, data = gaming_hear)

WRS2::yuen(GamingHabits_Comp ~ Group, data = gamers)
WRS2::yuen(GamingHabits_Video ~ Group, data = gamers)
```

```
#Remove Gaming==NA for gaming_wide object, for later steps of the analysis
gaming_wide_NA <- na.omit(gaming_wide[-9])

##Between-Between-Within robust ANOVA, restructure file and analysis##
gaming_wide_bbw2 <- bbw2list(gaming_wide_NA, grp.col = c(3, 4), lev.col=c(15, 16))
bbwtrim(2,2,2,gaming_wide_bbw2)

#Creating subsets for  calculation of confidence intervals on trimmed means
gaming_wide_deaf <- gaming_wide_NA[which(gaming_wide_NA$Group==1), ]
gaming_wide_deaf_gamer <- gaming_wide_deaf[which(gaming_wide_deaf$Gaming==1), ]
gaming_wide_deaf_nongamer <- gaming_wide_deaf[which(gaming_wide_deaf$Gaming==0), ]

gaming_wide_hear <- gaming_wide_NA[which(gaming_wide_NA$Group==2), ]
gaming_wide_hear_gamer <- gaming_wide_hear[which(gaming_wide_hear$Gaming==1), ]
gaming_wide_hear_nongamer <- gaming_wide_hear[which(gaming_wide_hear$Gaming==0), ]

#estimating confidence intervals
onesampb(gaming_wide_deaf_gamer$flanker_all_rt, est=mean, nboot=2000, SEED=TRUE, tr=0.2)
onesampb(gaming_wide_deaf_nongamer$flanker_all_rt, est=mean, nboot=2000, SEED=TRUE,
tr=0.2)
onesampb(gaming_wide_hear_gamer$flanker_all_rt, est=mean, nboot=2000, SEED=TRUE, tr=0.2)
onesampb(gaming_wide_hear_nongamer$flanker_all_rt, est=mean, nboot=2000, SEED=TRUE,
tr=0.2)
```
