# Supplementary material for: Evidence of an Effect of Gaming Experience on Visuospatial Attention in Deaf but Not in Hearing Individuals
Source: Front Psychol. 2020 Oct 20;11:534741. doi: 10.3389/fpsyg.2020.534741 (PMC7606995; doi:10.3389/fpsyg.2020.534741)
Supplement: Supplementary file 4 [file Data_Sheet_4.PDF]

List of PC/video games played by Gamers

Sorted by group and listed alphabetically (number of participants mentioning that game in parenthesis, if more than one)

Hearing participants

Assassin's Creed

Beyond: Two souls

Call of Duty
Civilization
Counter-Strike

Don't Starve Together
DOTA (2)
Dragon Age

Fallout
FIFA (3)
Fortnite

Grand Theft Auto

Halo (2)
Heavy Rain
Hitman
Hyperrogue

LEGO: The complete star wars saga
Life is Strange

Mario Kart
Mario & Luigi: Bowser Inside Story
Minecraft

Offworld Trading Company
Overcooked
Overwatch

PlayerUnknown's Battlegrounds (3)
Pro Evolution Soccer

Rust

Sims (2)
Singstar
Skylanders
Spider-Man (2)

Starcraft
Super Hexagon
Super Mario (3)

That’s You
The Vanishing of Ethan Carter
The witness
Tomb Raider

Undertale
Unravel

Warcraft

Deaf participants

Candy Crush Saga

Counter-Strike

Dark Souls

FIFA (4)

Grand Theft Auto

Killing Floor

Mario Kart
Minecraft
Monopoly

NHL

Risk

Sims
Star Wars
Super Mario (2)
Super Mario Party

Tetris
The Legend of Zelda: Breath of the wild
The Walking Dead

Uncharted 4 (2)

Watch Dogs
